# Short-term rewetting after summer drought does not alter soil fungal community composition and manganese peroxidase activity in managed temperate beech forests

**DOI:** 10.1371/journal.pone.0352444

**Published:** 2026-06-30

**Authors:** Shanza Zaib, Michaela Caboňová, Slavomír Adamčík, Marcel Zámocký, Jana Harichová, Zuzana Gajdošová, Katarína Adamčíková, Milan Valachovič, Richard Hrivnák, Tomáš Větrovský, Markus Gorfer, Rebecca Horváthová, Miroslav Caboň

**Affiliations:** 1 Plant Science and Biodiversity Centre, Slovak Academy of Sciences, Bratislava, Slovakia; 2 Laboratory of Phylogenomic Ecology, Institute of Molecular Biology, Slovak Academy of Sciences, Bratislava, Slovakia; 3 Department of Plant Pathology and Mycology, Institute of Forest Ecology, Slovak Academy of Sciences Zvolen, Nitra, Slovakia; 4 Institute of Microbiology of the Czech Academy of Sciences, Prague, Czech Republic; 5 Bioresources, AIT Austrian Institute of Technology GmbH, Tulln, Austria; 6 Department of Botany, Faculty of Natural Sciences, Comenius University in Bratislava, Bratislava, Slovakia; Institute for Sustainable Plant Protection, C.N.R., ITALY

## Abstract

Climate change is increasing the frequency of drought–rewetting cycles in temperate forests, yet the short-term responses of soil fungal communities to these events remain poorly resolved. We investigated whether an intense summer drought followed by heavy rainfall alters fungal community composition and oxidative enzyme activity in managed temperate beech forests of Central Slovakia. Soil samples from litter and mineral horizons were collected before and seven days after a rainfall event that followed an 18-day dry period. Fungal communities were characterized using ITS2 metabarcoding, and manganese peroxidase (MnPox) activity was measured as an indicator of oxidative decomposition potential. Fungal communities differed strongly between litter and mineral soil horizons across taxonomic and trophic classifications. Ectomycorrhizal fungi and soil saprotrophs dominated the mineral soil, whereas plant pathogens, litter saprotrophs, and wood-decomposing fungi were more abundant in the litter layer. In contrast, short-term rewetting did not produce detectable changes in fungal community composition or alpha diversity within either horizon. MnPox activity showed substantial spatial variability among plots but no consistent response to the rainfall event. Together, these results indicate that vertical stratification exerts a stronger influence on fungal community structure than short-term moisture fluctuations in these beech forest soils. The absence of rapid compositional change suggests that fungal communities in mature temperate forests may exhibit short-term resistance to drought–rewetting pulses, highlighting the importance of soil structure and litter layers in buffering microbial communities against transient climatic extremes.

## Introduction

Fungal communities are an important part of the soil microbiome, contributing to organic matter decomposition, nutrient cycling, and interacting with other organisms [1,2]. European beech (*Fagus sylvatica*) forests dominate large areas of Central Europe and host diverse soil fungal communities, which are key agents in the transformation of recalcitrant litter and the cycling of carbon and nitrogen [3]. These functions are often stratified across soil horizons. The litter layer, which is rich in freshly fallen leaves, supports primarily communities of decomposers, whereas the fragmented topsoil horizon, typically composed of more stabilized organic material, has a higher representation of symbiotic fungi and secondary decomposers [4–6]. Soil fungal communities of temperate forests (especially in monocultures) are directly or indirectly affected by ongoing climate change, because of increased risk of wildfires [7], shift in dominant mycorrhizal type among trees from ectomycorrhizal to arbuscular mycorrhizal [8], and changes in nutrient dynamics [9,10]. In the long-term, all the above-mentioned influences consequently result in mycobiome community turnover and further changes in soil enzymatic activities and nutrient dynamics [9,11].

Ongoing climate change is not only driving long-term warming but also increasing the frequency of extreme weather conditions, such as long droughts followed by high rainfalls, which can cause acute disturbances in fungal communities [12–14]. In Slovakia, a general trend towards increased drought severity has been observed, especially in spring and summer seasons [15]. Drought stress can induce changes in fungal community composition by favoring taxa that are better adapted to low moisture conditions [16]. It remains unclear whether a single post-drought rainfall pulse is sufficient to alter fungal community structure in mature forest soils, or whether communities exhibit short-term resistance. Additionally, drought conditions may reduce fungal biomass and suppress the production of extracellular enzymes in the litter and soil horizons [17,18], while sudden and intensive post-drought rainfalls can trigger rapid fungal growth and changes in community composition, the effects that may be temporary but otherwise significant for ecological studies [19,20].

Traditional approaches to study fungal communities (surveys, cultivations) proved to be challenging due to high variation in the seasonality, different fungal phenology and different mycelial growth and cultivation success of individual fungi [21]. Advances in high-throughput sequencing of short amplicons (so-called metabarcoding) enabled deeper insight into the composition of soil fungal communities by sequencing targeted short informative region yielding millions of sequences per sequencing run [22]. Considerable variation in the estimation of community composition could be introduced by different sampling strategies and sample processing, e.g., uneven horizon sampling [4,23], sample collection through longer period and seasonal community shifts [24], and due to improper sample handling and storage [23,25].

The present study investigates how intense summer drought conditions followed by sudden and intensive precipitation influence fungal community composition and functional activity across the litter and topsoil horizons in beech-dominated forests. Integrating enzymatic assays with molecular data enhances our ability to distinguish between structural and functional shifts, providing a more comprehensive view of soil fungal ecology under contemporary climate scenarios [26,27]. By combining DNA metabarcoding with enzymatic assays for manganese peroxidases activity as an indicator of lignin degradation and oxidative decomposition of recalcitrant organic compounds, we aim to: (1) evaluate the extent to which precipitation may alter fungal community structure and its ecological functions in distinct soil horizons, (2) assess whether manganese peroxidase activity varies in response to short-term climatic fluctuations.

## Materials and methods

### Study site and sampling

The study area was located in Central Slovakia, in the Javorie Mts. of volcanic origin with andesite bedrock [28]. The local climate is characterized by a mean annual precipitation of 831 mm and a mean annual temperature of 7.0 °C [29]. Soil sampling was conducted at two study areas previously described in detail by Hrivnák et al. [29]: 304a (center of the area: 48°33’16.2” N, 19°05’47.88” E, 345 m a.s.l.) and 504a (center of the area: 48°30’27.6” N, 19°07’39.9” E, 545 m a.s.l.). Both areas are situated on gentle north-facing slopes and are covered by a homogeneous, managed beech forest approximately 80 years old. As both sites were situated within managed forests and accessible through existing forest roads, no specific permissions to collect soil samples were needed. To eliminate the effect of different soil physico-chemical properties, both areas were selected to exhibit similar soil texture and properties, as measured by the AGES Institute. 304a: pH 4.97, plant available phosphorus 19 mg/kg, total organic carbon 3.98%, humus content derived from TOC analysis 6.8%, C/N ratio 15.73. 504a: pH 4.88, plant available phosphorus 17 mg/kg, total organic carbon 2.73%, humus content derived from TOC analysis 4.7%, C/N ratio 13.25. Plant available phosphate was determined spectrophotometrically after CAL-extraction and staining with molybdate following ÖNORM L 1087. Total organic carbon was measured upon dry combustion following ÖNORM L 1080. Humus was derived from TOC using the coeficient 1.7. pH was measured from water solution according to Fernandez et al. [30]

**Fig 1:**
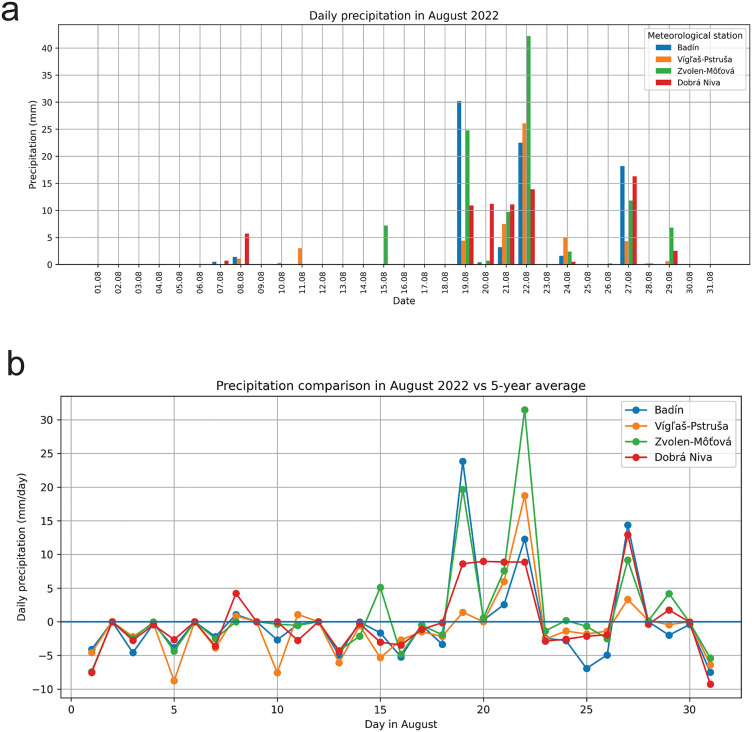
Precipitation dynamics in August 2022 at four closest meteorological stations surrounding the study area. **(a)** Daily precipitation data for August 2022 as measured at Badín, Vígľaš-Pstruša, Zvolen-Môťová, and Dobrá Niva. **(b)** Difference between observed daily precipitation in August 2022 and the corresponding 5-year daily average (data source: Slovak Hydrometeorological Institute).

Within each area (304a and 504a), two 5 × 5 m sampling plots at least 50 m apart, with homogeneous terrain (labeled as plots 1 and 2) were selected. Each plot was sampled on 18 August 2022 during the dry period and again on 25 August 2022 immediately after intensive rainfall (see Fig 1, Supplementary table 1). According to data from the nearest meteorological stations, the first sampling (18 Aug 2022) followed a prolonged period of generally low precipitation during the preceding 18 days. Although minor rainfall events occurred on 8 and 15 August, their magnitude was limited and did not substantially increase measured soil moisture at the time of dry sampling (Supplementary Table 1). A more intense rainfall period (>30 mm cumulative precipitation) occurred immediately before the second sampling on 25 Aug 2022. Each plot was sampled under both dry and wet conditions; therefore, dry and wet samples were treated as paired observations in statistical analyses.

At each plot, soil was sampled to a depth of 10 cm using five soil cores (surface sterilized PVC tube of 30 mm diameter) taken as subsamples: one from the center and four from the corners of the plot. In order to avoid cross-contamination, each sample was sampled using a separate clean, surface-sterilised and autoclaved set of tools. After collection, the organic litter horizon was separated from the underlying mineral topsoil. Subsamples from each horizon were pooled into a single composite sample. The topsoil samples were sieved through a 4 mm mesh, and all visible organic debris (plant litter, roots, insects, etc.) was manually removed [31,32]. Both the organic horizon and topsoil samples were immediately after sampling and debris removal stored in liquid nitrogen in the field and transported to the laboratory for further analysis, where they were kept at −80°C until processed. For each composite sample, we measured pH and moisture from the soil horizon in three technical repetitions and provided average values (see Supplementary table 1).

The following labeling is used in the manuscript: samples collected during the dry period are referred to as dry (D) moisture condition, and those collected after rainfall as wet (W) condition. The upper organic litter layer is referred to as litter (L), and the underlying topsoil up to 10 cm as soil (S) horizon. Based on the combination of sampling period and horizon, samples were assigned to four categories: LD (litter, dry), LW (litter, wet), SD (soil, dry), and SW (soil, wet).

### Enzymatic activity analyses

Manganese-peroxidase (MnPox) was measured to estimate fungal ligninolytic activity, these enzymes are fungal-specific and are strongly related to oxidative decomposition of lignin and carbon dynamics [33]. MnPox activity was measured in three repetitions per sample only from S horizon as suggested by Packard et al. [34] and measured at the wavelength of 238 nm on the CaryUVvis 60 spectrophotometer from Agilent (Santa Clara USA) according to the original protocol by forming a Mn3 + -malonate complex in the presence of H_2_O_2_ [35]. Enzyme activities were measured at a constant temperature of 25°C in 3mL quartz cuvettes. Soil samples were first homogenized in 100 mM sodium phosphate buffer (pH 7.0) with 1 mm glass beads. Afterward, they were centrifuged at 10 000 × g at 4°C for 15 minutes and the obtained supernatant was used for recording the manganese peroxidase activity in at least three technical replicates. For the calculation of MnPox activity, the ε 238nm = 6.5 mM-1 cm-1 was used [36] and 1 enzyme unit (1U) was defined as the oxidation of 1µmol Mn^2+^ cations to Mn^3+^ in 1 minute under standard assay conditions. Obtained values were calculated per g of each soil sample. For comparison of specific activities in different samples the Bradford method [37] of protein concentration determination was applied by using the Serva Bradford solution (Heidelberg, Germany). Prior to usage, 5x Bradford solution concentrate was diluted 1:4 with H_2_O and filtered according to manufacturer ‘s recommendations. Absorbance was recorded at 595 nm on CaryUVvis 60 spectrophotometer. For the calibration curve bovine serum albumin in the concentration range 0.1/1.0 mg/ml was used.

### DNA amplification and sequencing

Environmental DNA (eDNA) from either soil or litter samples was extracted in three replicates following the modified cetyl trimethylammonium bromide (CTAB) protocol of Sagová-Marečková et al. [38]. eDNA samples were purified using MoBio PowerClean DNA extraction cleanup kit and pooled into a single composite sample. Sample quality was determined by Shimadzu Spectrophotometer and eDNA was quantified using Qubit Broad Range kit. Concentration of eDNA samples was adjusted to a uniform concentration 5 ng/µl by dilution with sterilised deionised water. The fungal soil community was estimated by metabarcoding analysis of the internal transcribed spacer 2 region of the ribosomal DNA operon (ITS2 rDNA) using modified ITS3F and ITS4R primers [39]. PCR amplification, library preparation and amplicon sequencing were performed by a commercial sequencing company SEQme s.r.o. (Dobříš, Czech Republic) on Illumina NovaSeq using NextTerra reagent kit, following standard protocols used by the company. All samples were sequenced in a single Illumina run.

### Sequence data processing

Amplicon sequence data were processed using programs implemented in the SEED 2.0.1. pipeline [40]. Sequences were pair-joined using fastq-join v. 1.1.2 [41] with 15% maximum difference and minimum overlap of 40 bp and further quality checked, leaving only sequences with phred score (qm) higher than 30. ITS2 region was retrieved using ITSx v. 1.0.11 with settings to retrieve complete fungal regions only [42]. Prior to taxonomic assignment, sequences were clustered into operational taxonomic units (OTUs) using UPARSE in USEARCH v 11.0.667 [43], applying a 97% similarity threshold. A 97% similarity threshold was used to maintain comparability with previous forest fungal studies and to reduce artificial inflation of diversity due to intragenomic ITS variability. Chimeric sequences and low-abundant OTUs (fewer than 4 sequences per OTU) were removed. Sequences of Illumina reads were deposited in the Sequence Read Archive in BioProject PRJNA1321012 under accession numbers SAMN51193650– SAMN51193665.

### Taxonomic and trophic mode assignment

The most abundant sequence in each OTU in the ITS2 dataset was compared against the UNITE fungal ITS reference database release 9.0 [44,45]. Additionally, all non-fungal OTUs and OTUs not exceeding 0.1% relative abundance in at least one sample were excluded. Fungal ecological guilds were initially assigned to ITS2 OTUs by the FungalTraits database [46]. For simplification, identified guilds were grouped into eight major categories: (1) ectomycorrhizal fungi (EcM), (2) litter saprotrophs, (3) plant pathogens, (4) other pathogens and parasites (including lichen parasites, animal parasites, mycoparasites), (5) other saprotrophs (including soothy molds, dung saprotrophs, unspecified saprotrophs), (6) other symbiotrophs (including foliar endophytes, arbuscular, ericoid- and orchid mycorrhiza and lichens), (7) soil saprotrophs and (8) wood saprotrophs. Taxonomic units without any defined guilds are classified as not defined (ND).

### Statistical analyses

Raw sequence reads were transformed into relative read abundance (RRA), which is expressed as the proportion of sequencing reads assigned to a given taxon relative to the total reads in a sample after quality filtering and taxonomic assignment. Mean relative read abundances are reported with standard deviations. For statistical analyses, dataset of reads transformed to relative read abundance with only units exceeding 0.1% in at least single sample was used. Alpha diversity indices (OTU richness, Shannon diversity, Simpson 1–D, Inverse Simpson, Evenness, and Dominance) were calculated from relative abundance OTU tables. Observed richness was computed as the number of OTUs with non-zero abundance per sample. Shannon, Simpson (1–D), and Inverse Simpson indices were calculated following standard formulations based on relative abundances. Because dry and wet samples were collected from the same plots, moisture represented a within-plot factor. To account for repeated sampling, alpha diversity responses were analysed using linear mixed-effects models with moisture and horizon as fixed effects and plot included as a random factor. To quantify the magnitude of moisture-induced changes independently of statistical significance, paired effect sizes were calculated for each horizon separately. For each plot, the difference between wet and dry conditions (Δ = Wet − Dry) was computed. Mean paired differences, standard deviations of the differences, 95% confidence intervals, and Cohen’s d for paired samples were calculated. Effect sizes were interpreted using conventional thresholds (small ≈ 0.2, moderate ≈ 0.5, large ≥ 0.8).

Differences in fungal community composition between moisture conditions were evaluated using permutational multivariate analysis of variance (PERMANOVA) based on Bray–Curtis dissimilarities calculated from OTU relative abundance data. Homogeneity of multivariate dispersion was assessed prior to interpretation. To respect the repeated-measures design and avoid violations of permutation exchangeability, PERMANOVA was conducted separately within each horizon (litter and soil). Within each horizon, moisture condition (dry vs wet) was used as the explanatory factor, and significance was assessed using 9999 permutations. Effect sizes were quantified using the proportion of variance explained (R²).

Both ordination analysis Non-metric multidimensional scaling (NMDS) and Principal Coordinates Analysis (PCoA) were calculated based on Bray-Curtis dissimilarity. In order to maintain simple graphical visualisation, barcharts of relative read abundance (RRA) are presented as average values per sampling area or per moisture condition. To identify taxa associated with specific horizons or moisture conditions, indicator analyses were perfomed on relative abundances using the ‘multipatt’ function (func = “IndVal.g”) with 999 permutations and p < 0.05 [47]. The effect of moisture treatment (dry vs. wet) on MnPox activity was tested using a linear model. Statistical significance was evaluated using analysis of variance (ANOVA). All statistical analyses and graphical visualizations were performed in RStudio 2023.06.0. using packages: tidyverse [48], vegan [49], ggpubr [50], lmerTest [51] pairwiseAdonis [52], dplyr [53], ggplot2 [54], pheatmap [55], tibble [56], indicspecies [57].

## Results

### Total fungal diversity

All 16 samples were successfully sequenced and yielded on average 54938 ± 13463 of quality-filtered and pair-joined reads. Clustering resulted in 1823 OTUs, of which 593 OTUs were retained applying 0.1% relative abundance threshold. Twelve OTUs occurred in at least 10 samples, and the most abundant OTU (*Pezicula* sp.) was detected in all samples. The number of OTUs per sample ranged 227–347 (avg. 298.4 ± 33.8), with slightly lower average values in samples from S horizon (Supplementary table 2).

**Fig 2:**
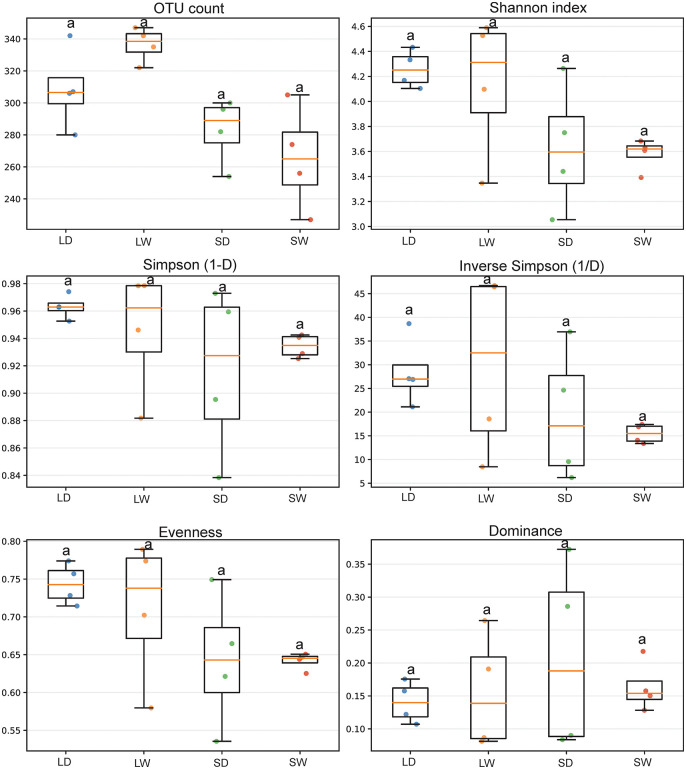
Alpha-diversity of communities across horizons and moisture conditions. Panels show six indices: OTUs count, Shannon (H′), Simpson diversity (1–D), Inverse Simpson (1/D), Pielou’s evenness (J′), and Simpson dominance **(D)**. Boxplots display the median (line), interquartile range (box), and 1.5 × IQR whiskers; black points indicate values of individual samples. Differences in alpha-diversity indices among samples were tested using the non-parametric Kruskal–Wallis test. As no significant differences were detected (p ≥ 0.05), all groups were considered statistically homogeneous. Abbreviations: LD litter in dry condition, LW litter in wet condition, SD soil in dry condition, SW soil in wet condition.

Alpha diversity indices (OTU richness, Shannon diversity, Simpson 1–D, Inverse Simpson, Evenness, and Dominance) differed primarily between horizons, whereas no consistent differences were observed between moisture conditions (Fig 2). Paired comparisons within plots showed no consistent Dry vs. Wet directional shifts in alpha diversity within either horizon (Supplementary figure 1). Across all six indices, trajectories varied among plots but did not show a uniform Dry vs. Wet shift. PERMANOVA analyses conducted separately within each horizon revealed no significant effect of short-term rewetting on fungal community composition in either horizon. In litter, moisture explained 14.3% of variation (F = 0.997, p = 0.138), whereas in soil it accounted for 5.0% (F = 0.314, p = 0.127). These results indicate that compositional shifts associated with short-term rewetting were small relative to between-plot variation.

**Fig 3:**
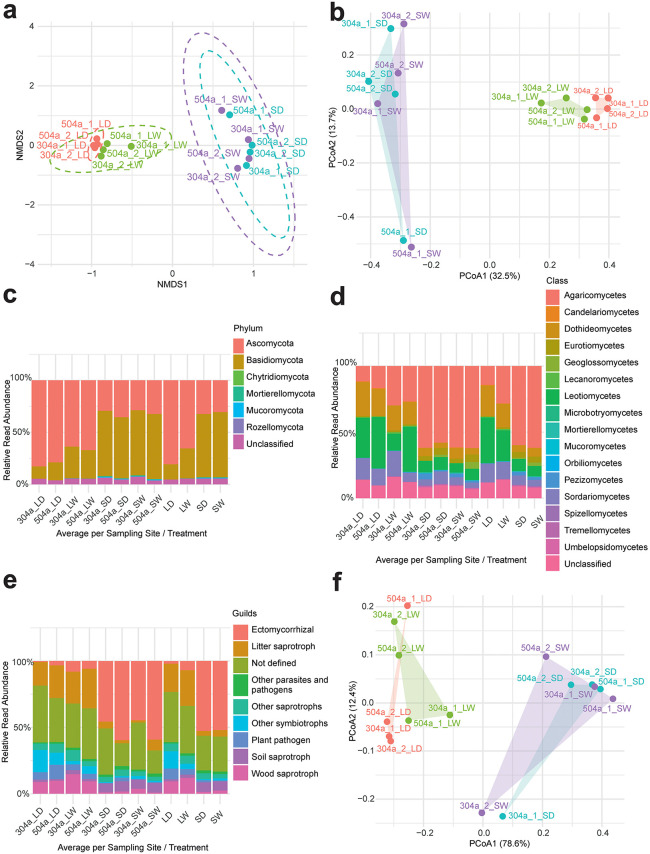
Statistical analyses of alpha and beta diversity performed on samples from different horizons and moisture conditions. **a** Non-metric multidimensional scaling (NMDS) ordination of the whole fungal community based on Bray-Curtis Dissimilarity with 0.95 confidence ellipses. **b** Principal Coordinate Analysis (PCoA) of the whole fungal community. Percentage of variation for each axis is indicated within brackets. **c–e** Stacked charts of mean relative fungal read abundance sorted by phylum **(c)**, class (d) and guild **(e)**. For each site average values of 2 plots are presented and for each moisture condition, the average of 4 plots from both sampling sites. **f** PCoA analysis based on analysis of relative read abundance of detected fungal guilds. Percentage of variation for each axis is indicated within brackets. Abbreviations: LD litter in dry condition, LW litter in wet condition, SD soil in dry condition, SW soil in wet condition.

NMDS and PCoA ordinations showed clear separation between horizons but no separation between moisture conditions within horizons (Fig 3a–b). NMDS showed clear separation of samples from different horizons, but samples of different moisture conditions do not differ within each horizon. PCoA analysis showed the same pattern of significant differences between horizons but not between moisture conditions.

### Stratification of soil fungal communities across horizons

On average, 82.9 ± 10.4% of fungal reads were identified at the order level and 66.6 ± 13.5% at the genus level (Supplementary table 2). At both phylum and class levels, no moisture-driven shifts were detected within either horizon, whereas strong separation between horizons was consistently observed (Fig 3c–d). In terms of taxonomic identification at the phylum level, both LD and LW were dominated by Ascomycota (80.8 ± 6.2% and 65.5 ± 7.1% respectively) followed by Basidiomycota (LD – 14.6 ± 6.4%, LW – 28.8 ± 7.4%). Soil horizons showed an opposite pattern, with Basidiomycota dominating as the most abundant phylum (SD – 60.8 ± 3.7%, SW – 62.6 ± 8.2%) followed by Ascomycota (SD – 32.4 ± 5.5%, SW – 30.7 ± 5.2%). All other phyla were represented by less than 1% of RRA proportions.

Strong differences between L and S horizons were observed also at the class level in which LD was predominantly colonized by Leotiomycetes (34.6 ± 5.2%) followed by Dothideomycetes (22.9 ± 6.1%) and Agaricomycetes (14.2 ± 6.4%). LW samples were dominated by Agaricomycetes (28.4 ± 7.4%), followed by Leotiomycetes (23.3 ± 12.3%) and Dothideomycetes (17.9 ± 1.8%). The only other group represented by more than 5% of RRA was Sordariomycetes (LD –14.2 ± 2.7%, LW – 13.1 ± 8.4%). Soil samples were predominantly colonized by Agaricomycetes (SD – 59.9 ± 3.7%, SW – 61.9 ± 8.2%). Only four other classes reached in average more than 5% of RRA proportion in at least one sample: Dothideomycetes, Eurotiomycetes, Leotiomycetes and Sordariomycetes.

### Trophic mode analysis

Clear differences in fungal guild composition were observed between litter and soil horizons, whereas dry and wet samples within the same horizon showed largely overlapping patterns. Soil samples (SD and SW) displayed similar guild structures to each other but contrasted strongly with litter samples (LD and LW). Likewise, litter communities were more similar between dry and wet conditions than to any soil samples. These patterns indicate that vertical stratification, rather than short-term precipitation, was the primary driver of guild composition. Although minor shifts in relative abundances were visible in the bar charts following rainfall, these changes did not represent a consistent restructuring of guild representation within horizons over the short sampling interval. (Fig 3e).

**Fig 4:**
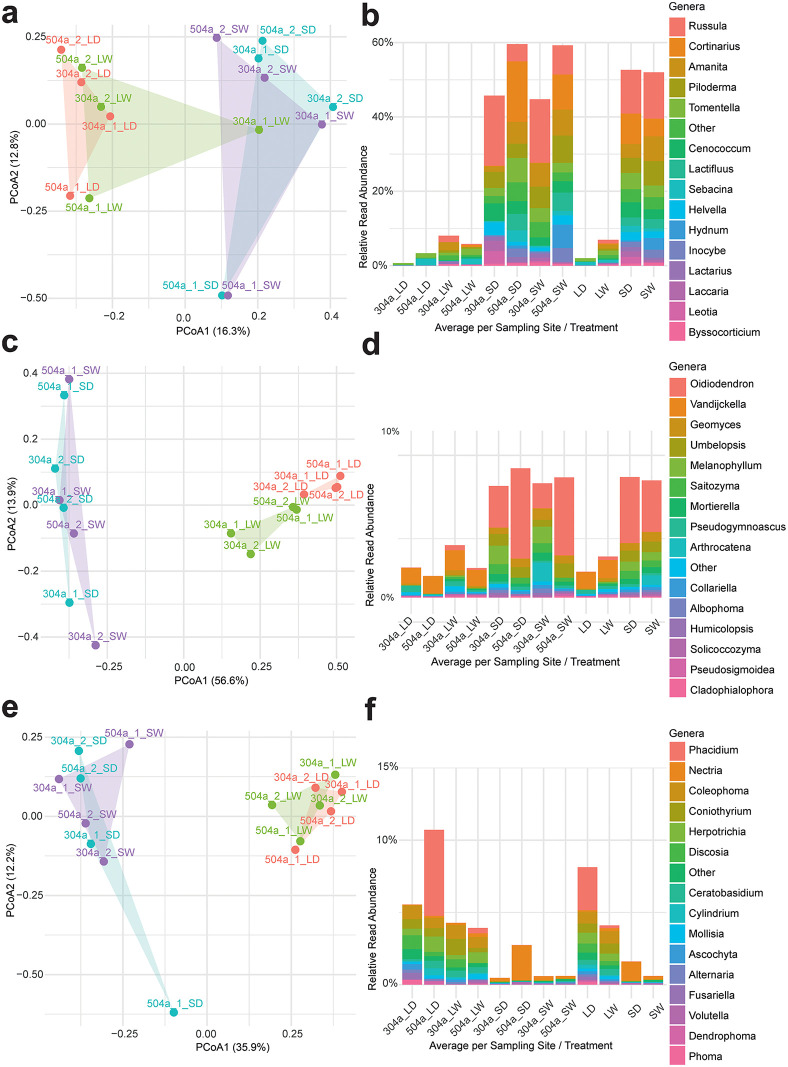
Community compositions within selected fungal guilds. Left: Principal Coordinate Analysis based on Bray-Curtis dissimilarity. Right:stacked charts (right) showcasing 15 most abundant fungal genera within selected fungal guild. **a–b** Ectomycorrhizal fungi. **c–d** Soil saprotrophs. **e–f** Plant pathogens. Abbreviations: LD litter in dry condition, LW litter in wet condition, SD soil in dry condition, SW soil in wet condition.

When evaluating the most abundant fungal guilds individually, each displayed the same trend of clear separation between horizons, but not between moisture conditions. Ectomycorrhizal fungi (Fig 4A and 4B), represented by 116 OTUs, largely dominated in S horizon comprising 52.7 ± 15.5% of all reads in SD and 52 ± 18.8% in SW, with the most abundant genera *Russula*, *Cortinarius*, *Amanita*, *Piloderma* and *Tomentella*. Their abundance was significantly lower in the litter horizon, with 2.02 ± 1.8% in LD and 6.9 ± 5.6% in LW. Among all analysed fungal guilds, ectomycorrhizal fungi showed pronounced differences in relative abundance between litter and soil horizons, dominating the mineral soil while remaining rare in litter, even though this pattern was not entirely mirrored by PCoA analysis (Fig 4a). Soil saprotrophs were consistently more abundant in in S horizon (SD: 7.22 ± 1.82%; SW: 7.02 ± 2.34%) compared to litter (LD – 1.55 ± 0.33%; LW – 2.46 ± 1.45%) (Fig 4D). Soil saprotrophs showed minor separation between LD and LW in ordination space, although their relative abundance remained low (<10.1%). The most abundant genera of soil saprothrophs were *Oidiodendron* in S samples and *Vandijckella* in L samples (Fig 4C).

**Fig 5:**
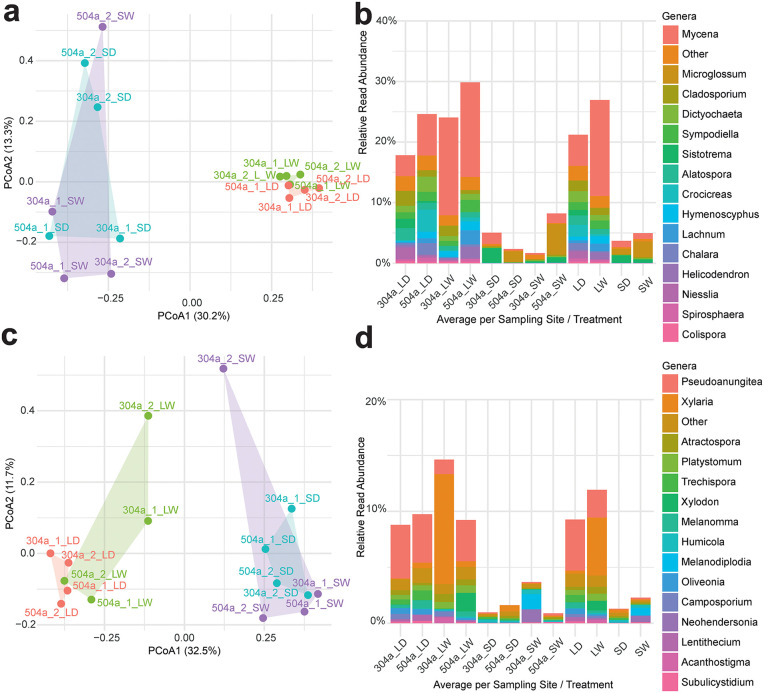
Community compositions within selected fungal guilds. Left: Principal Coordinate Analysis based on Bray-Curtis dissimilarity. Right:stacked charts (right) showcasing 15 most abundant fungal genera within selected fungal guild. **a–b** Litter saprotrophs **c–d** Wood saprotrophs. Abbreviations: LD litter in dry condition, LW litter in wet condition, SD soil in dry condition, SW soil in wet condition.

Other analysed fungal guilds showed contrasting vertical patterns, being more dominant in the litter horizon. Litter saprotrophs, plant pathogens, and wood saprotrophs were consistently more abundant in litter than in soil, while differences between moisture conditions were negligible. Litter saprotrophs were more abundant in LW (26.9 ± 7.1%) and LD (21.2 ± 4.2%), while being rare in soil (Fig 5a–b). This guild included 71 OTUs, and the genus *Mycena* was the most dominant across all litter samples. Similarly, Wood saprotrophs were more represented in L horizons (LW – 11.9 ± 7.6%, LD – 3 ± 1.3%), but were nearly absent in S (Fig 5c–d). This guild comprised of 55 recognized OTUs, and genera *Pseudoanungitea* and *Xylaria* were the most abundant in the L samples. Representation of plant pathogens was generally low across all samples with higher abundance in L horizon (LD – 8.13 ± 4.17%, LW – 4.1 ± 0.43%) (Fig 4e–f). Among 31 recognized OTUs of this guild, the most dominant genera were *Phacidium*, *Nectria* and *Coleophoma*.

### Analysis of indicator species

**Fig 6:**
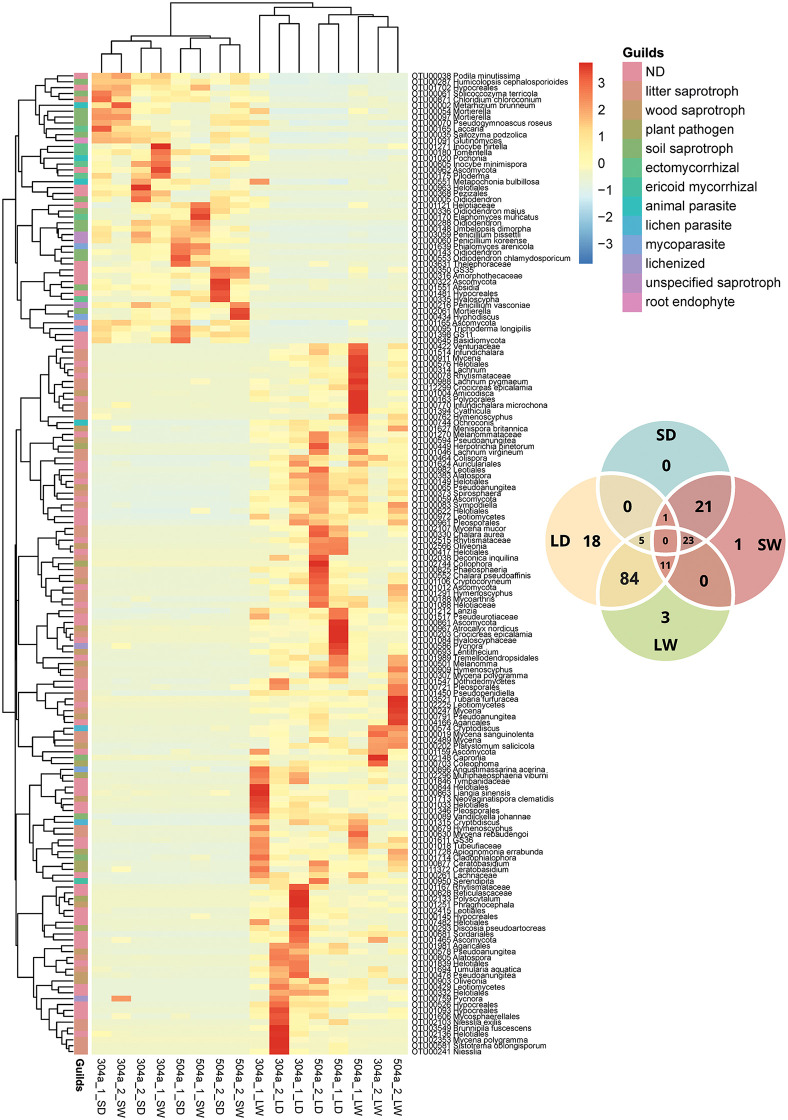
Heatmap showing indicators (in row) at OTU-level and their trophic mode assignments for each sample (in columns). Colors show row-scaled relative abundance (z-scores; −3 to +3). The left color bar annotates each OTU’s ecological guild (legend at right). Dendrograms indicate hierarchical clustering of taxa and samples. Insert Venn diagram shows numbers of taxa unique for each horizon and moisture combinations and shared among them. Abbreviations: LD litter in dry condition, LW litter in wet condition, SD soil in dry condition, SW soil in wet condition.

At the species (OTU) level, 167 indicators were identified, with only a few OTUs indicated single combination of horizon and moisture condition specifically (LD: 18, LW: 3, SD: 0 and SW: 1) (Supplementary table 3). Most indicators were shared either among litter horizons (84 OTUs) or among soil horizons (21 OTUs). Heatmaps combined with clustering analysis consistently also revealed clear differences between litter and soil samples. No OTUs were exclusively associated with moisture condition irrespective of horizon (Fig 6).

The majority of S horizon indicators with defined guilds belonged to soil saprotrophs and ectomycorrhizal fungi. On the contrary, indicators of L horizon were predominantly litter or wood saprotrophs. An additional indicator analysis at the genus level was conducted for five primary fungal guilds. This analysis identified 41 indicator genera, including plant pathogens, wood saprotrophs, soil saprotrophs, and litter saprotrophs. No ectomycorrhizal genera met the statistical threshold to be considered indicators.

**Fig 7:**
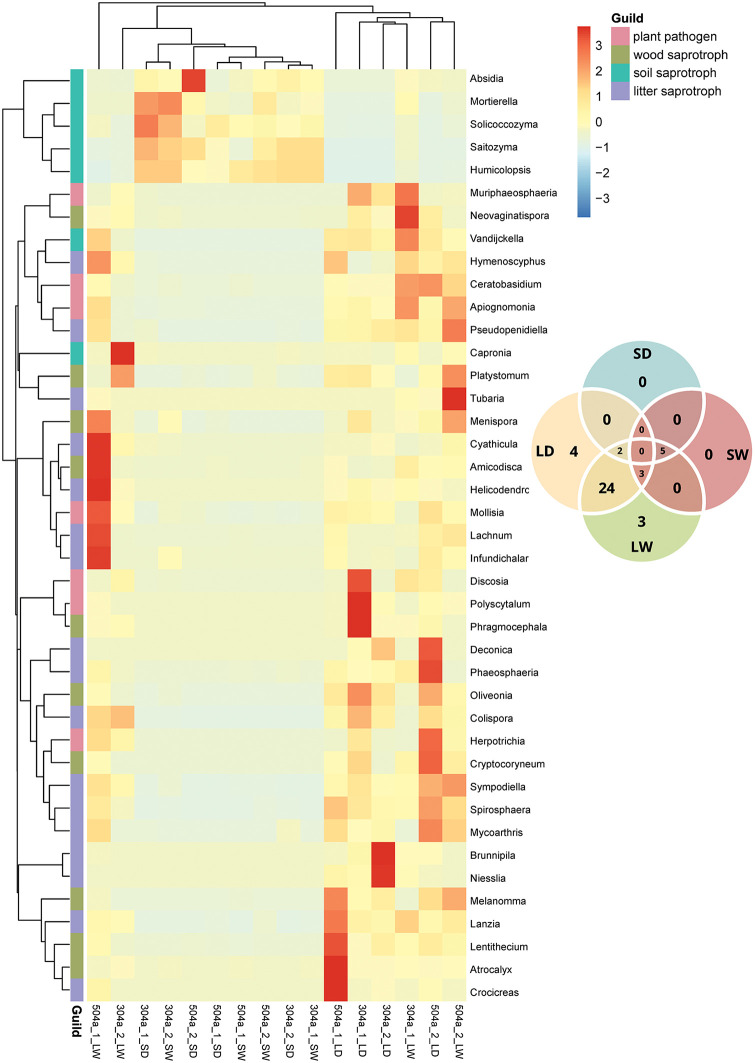
Heatmap showing indicators (in row) at genus-level and their trophic mode assignments for each sample (in columns). Colors show row-scaled relative abundance (z-scores; −3 to +3). The left color bar annotates each genus’s ecological guild (legend at right). Dendrograms indicate hierarchical clustering of genera and samples. Inset Venn diagram shows numbers of genera unique for each horizon and moisture combinations and shared among them. Abbreviations: LD litter in dry condition, LW litter in wet condition, SD soil in dry condition, SW soil in wet condition.

At the genus level, no indicators exclusively associated with samples of S horizon were detected (Fig 7). Within litter samples, four indicator genera *Polyscytalum*, *Phragmocephala*, *Deconica*, *Brunnipila* were identified for LD, and three genera *Amicodisca*, *Tubaria*, *Capronia* for LW. The majority (24 genera) were shared across both LD and LW moisture conditions.

### Enzymatic activity

**Fig 8:**
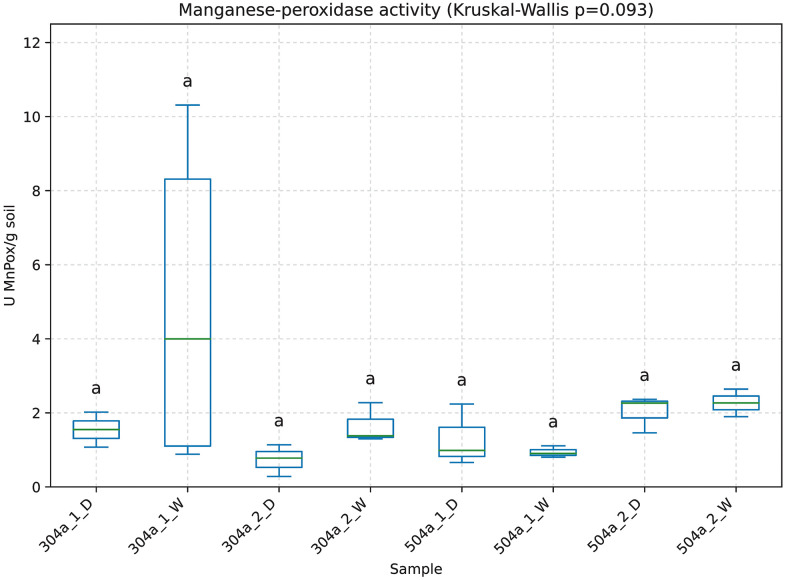
Manganese-peroxidase (MnPox) activity in the S horizon of the four plots during dry (D) and wet (W) condition. Boxplots display the median (line), interquartile range (box), and 1.5 × IQR whiskers. Differences in manganese-peroxidase activity among samples were tested using the non-parametric Kruskal–Wallis test. As no significant differences were detected (p ≥ 0.05), all groups were considered statistically homogeneous. Abbreviations: LD litter in dry condition, LW litter in wet condition, SD soil in dry condition, SW soil in wet condition.

Manganese-peroxidase activity, measured in U MnPox/g soil, did not differ significantly between moisture regimes (ANOVA, F = 1.87, p = 0.185) and showed no significant variation among plots (F = 1.92, p = 0.156), although variability among samples suggests potential influence of site-specific conditions. (Fig 8). A general trend of increased specific MnPox activity was observed under wet conditions, with the exception of sample 304a_1_W, which showed reduced activity compared to its dry counterpart 304a_1_D. However, this pattern was not observed in the second plot from the same site (304a_2), indicating that the anomaly may be due to local microhabitat variability or other site-specific factors. Although mean MnPox activity was numerically higher under wet conditions in most plots, this trend was not statistically supported.

## Discussion

### Changes in soil fungal community composition

Our results show that vertical stratification explained more variation in fungal community structure than short-term post-drought rewetting in these temperate beech forests. Across all taxonomic levels and functional guilds, litter and mineral soil communities remained clearly separated, whereas moisture explained only a small proportion of variance within each horizon. In forest soils, litter layers and soil structure may further buffer desiccation stress by retaining microsites of moisture [57]. Therefore, the fungal communities investigated here may be adapted to intermittent summer drying–rewetting cycles, resulting in short-term resistance of community composition despite changes in precipitation. Our samples were collected before and seven days after an intense rainfall event following an 18-day relatively dry period, allowing us to evaluate short-term compositional responses under realistic field conditions. Within this temporal window, no statistically detectable shifts in overall community composition or alpha diversity were observed. Given the limited number of independent plots, subtle treatment effects cannot be excluded, and larger-scale replication would be required to fully resolve small compositional shifts. Despite this limitation, our results suggest limited short-term compositional sensitivity to a single rewetting event. Mucha et al. [58] and Danzberger et al. [59] showed that various fungal guilds in temperate beech forests have different responses towards irrigation after long-term drought exposure over a longer period of 3 months. This suggests that fungal community responses may require longer or repeated moisture perturbations to become detectable, which could explain the absence of significant compositional changes observed in our short-term sampling window. Reports from beech forests contrast with reports from grassland and agricultural systems, where even short-term drought and rewetting cycles can drive significant changes in fungal community structure [60]. The relative stability observed in our study may reflect ecosystem-specific factors, such as established forest canopy, a relatively thick layer of litter horizon covering the soil, and the buffering water retention capacity of soil horizon, which may (as also suggested by Mucha et al. [58] moderate environmental fluctuations and reduce the intensity of microbial responses. Because our initial sampling followed a naturally occurring dry spell rather than an experimentally imposed continuous drought, our findings should be interpreted as responses to short-term field moisture dynamics under realistic summer conditions rather than as a strict drought-rewetting experiment.

We did not observe statistically significant moisture-driven shifts in taxonomic or guild fungal community composition. In contrast, several studies from boreal and temperate ecosystems have reported fungal community turnover under prolonged moisture changes or seasonal transitions (e.g., [61–65]), even though proportions of reads related to certain fungal guilds remained stable.

We recognized ectomycorrhizal fungi as the most dominant fungal guild in S horizon, but we did not identified any genera indicating differences in moisture conditions. The lack of detectable short-term shifts in ectomycorrhizal abundance may reflect a combination of their strict host association, soil water retention capacity, and physiological adaptations that protect them from short-term moisture disturbances [64,65]. Studies involving longer drought treatments or experimental irrigation have reported ectomycorrhizal shifts, suggesting that response magnitude depends strongly on treatment duration [66].

Soil saprotrophs, while less abundant than ectomycorrhizal fungi, also showed consistent presence in S horizon and were similarly unaffected by moisture conditions. This suggests a close association with more recalcitrant soil organic matter pools, which change slowly and may provide stable substrate humidity independent of surface conditions [33,67,68]. Soil saprotrophs showed minor compositional separation between dry and wet litter samples, although their relative abundance remained low. Our findings are consistent with studies emphasizing the importance of long-term substrate availability and stability in shaping microbial decomposer communities [68,69].

In L horizon, litter saprotrophs, wood saprotrophs, and plant pathogens were significantly more abundant. These fungi showed numerically higher relative abundance after rainfall, particularly in the case of litter saprotrophs. However, their taxonomic composition at the genus level remained relatively similar, suggesting that short-term moisture changes influence fungal activity or biomass rather than community turnover. This is congruent with studies of Mucha et al. [53] and Danzberger et al. [59] who found that fungal biomass fluctuated with drought and rewetting events, without change in the taxonomic composition.

The differences in composition of fungal soil communities in temperate beech forests among L and S horizons observed in this study were consistent across all detected major fungal guilds and were not influenced by changes in moisture conditions. This agrees with earlier findings that soil depth and subsequent changes in soil physio-chemical properties (organic matter content, C:N proportion) play important roles in shaping soil fungal communities [9,70–73].

### Changes in enzymatic MnPox activities

Manganese peroxidases are primarily produced by ligninolytic fungi, particularly basidiomycetes [74] and they are crucial for the oxidative degradation of lignin, which is a major structural component of leaf litter in beech forests [4,75]. Although fungal community composition in the soil horizon did not differ between moisture conditions, MnPox activity was numerically higher in wet samples in most plots. Supporting our observations, Criquet et al. [76] reported MnPox activity only during humid periods in Mediterranean evergreen oak forests. Observed variation in MnPox activity may reflects shifts in microbial metabolic priorities under different moisture conditions [69,77]. We did not observe links between MnPox activity and shifts in ectomycorrhizal or saprotrophic relative abundance, suggesting that short-term functional dynamics may be decoupled from taxonomic composition. This decoupling is consistent with the idea that functional responses can occur through physiological adjustment within stable communities rather than through community turnover. High within-site variability found in our study highlights the spatial heterogeneity of enzymatic processes in forest soils and complicates direct links between community composition and functional potential. Our first hypothesis, that vertical stratification would explain more variation than short-term rewetting, was supported. In contrast, our expectation that oxidative enzyme activity might respond rapidly to moisture pulses was not supported under the short temporal window examined.

## Conclusions

Our study demonstrates that vertical stratification is a stronger determinant of fungal community structure than a single short-term rewetting event in temperate beech forests of Central Slovakia. As with all metabarcoding studies, interpretation of relative read abundance is subject to methodological limitations [78–82]. Sampling design and habitat-specific characteristics likely influence the magnitude of detectable community responses. These processes require further investigation, particularly because the timescales over which fungal communities and enzymatic activities respond to climatic fluctuations are also affected by other factors such as seasonality or habitat heterogeneity [25,83,84]. Forest soils exhibit pronounced spatial heterogeneity in both community composition and enzymatic activity [85]. Such variability was evident even within sites exposed to identical climatic conditions, as illustrated by the contrasting MnPox activities of samples 304_1_W and 304_2_W. This highlights the necessity of sufficient replication when studying the functional structure of soil fungal communities. Our study is based on four independent plots, and therefore, the statistical power to detect subtle treatment effects is limited. While the repeated-measures design strengthens within-plot comparisons, broader generalizations should be made cautiously. Future study incorporating larger spatial replication and longer-term temporal monitoring would allow more robust evaluation of fungal responses to hydrological extremes.

## Supporting information

S1 Table Moisture level of soil horizon during Dry period (18 Aug 2022) and after intense precipitation (25 Aug 2022). (XLSX)

S2 Table List of OTUs with abundance 0.1% in at least single sample; their taxonomic identification, trophic mode assignment and matrix of relative read abundance in the samples. (XLSX)

S3 Table List of OTUs and genera identified as indicators. (XLSX)

S4 Table Measurements of manganese-peroxidase activity. (XLSX)

S1 Fig Paired changes in fungal diversity indices between dry and wet conditions. (PDF)
