# Supplementary figures and images for: Short-term rewetting after summer drought does not alter soil fungal community composition and manganese peroxidase activity in managed temperate beech forests
Source: PLoS One. 2026 Jun 30;21(6):e0352444. doi: 10.1371/journal.pone.0352444 (PMC13318017; doi:10.1371/journal.pone.0352444)

304a\_1 304a\_2 504a\_1 504a\_2

Horizon L

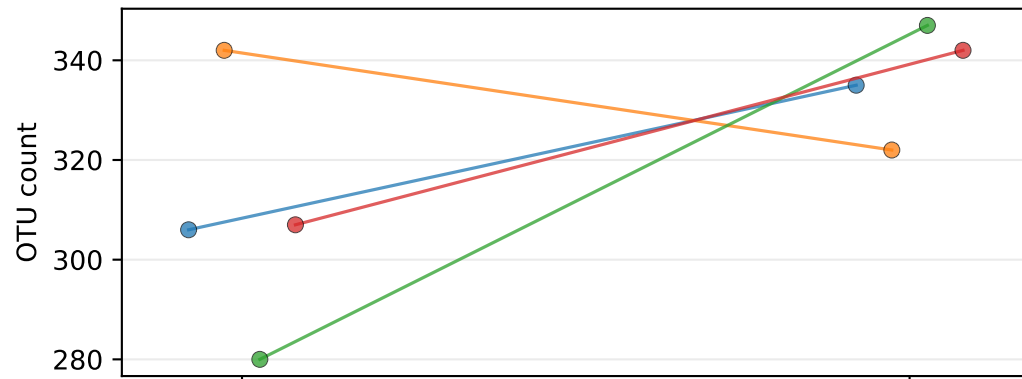

Horizon S

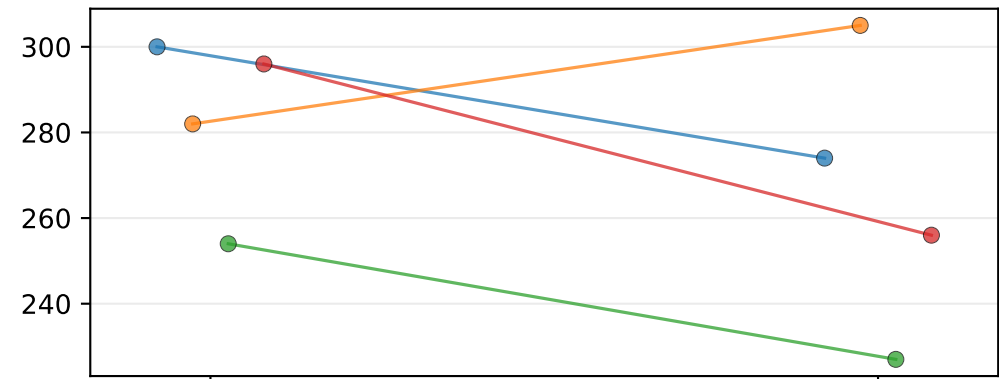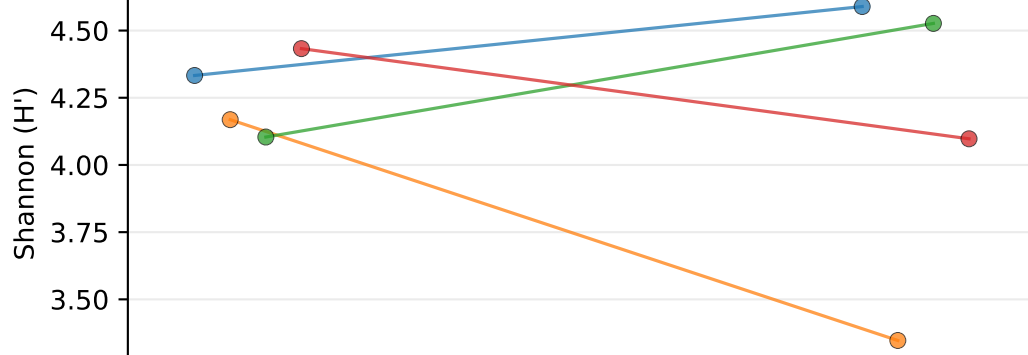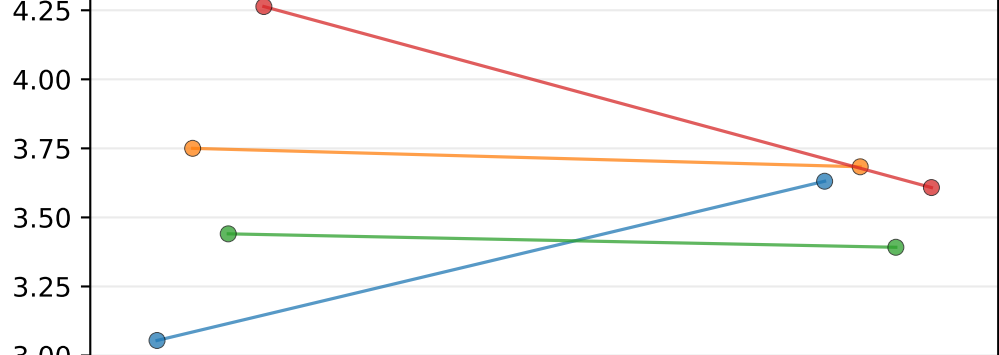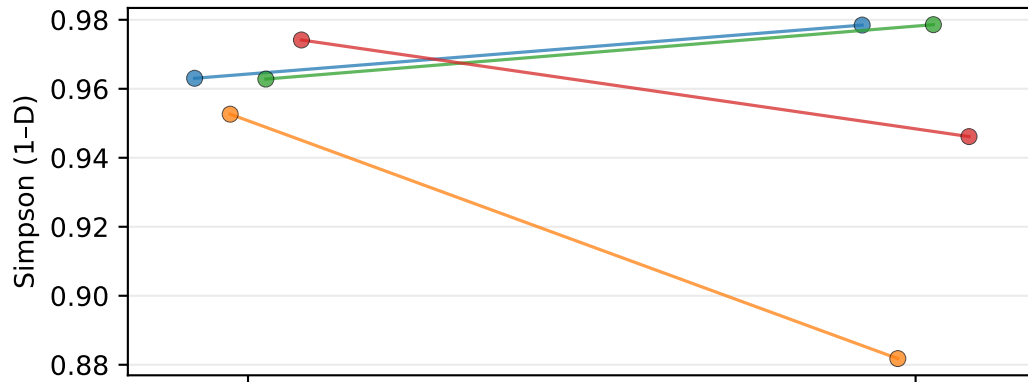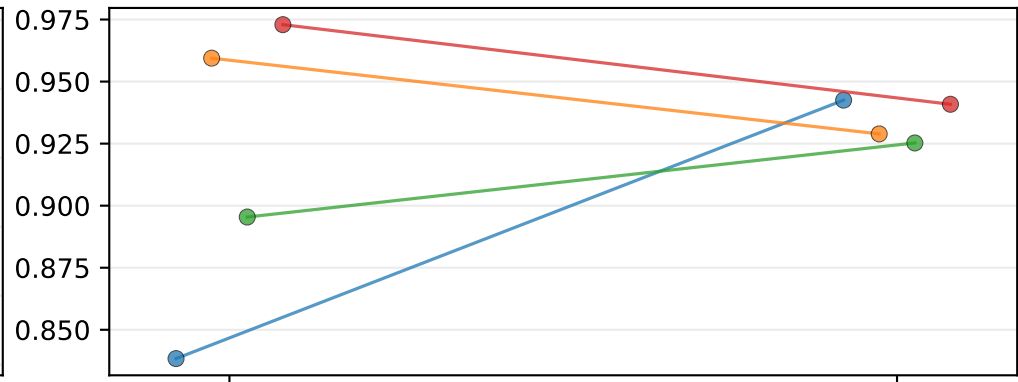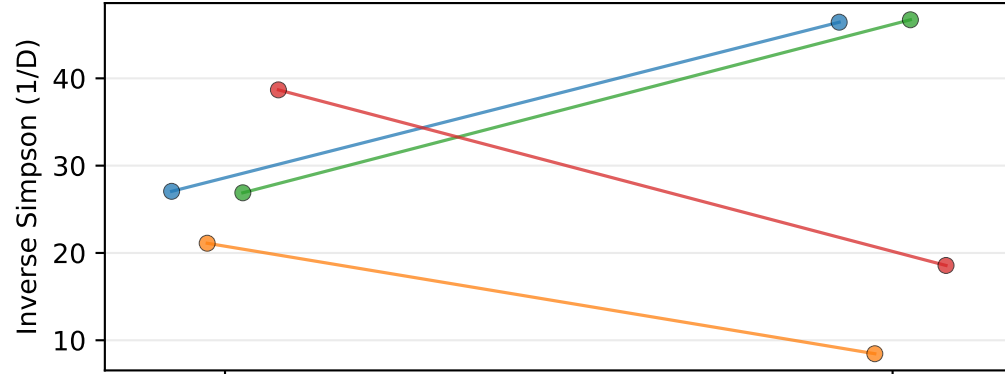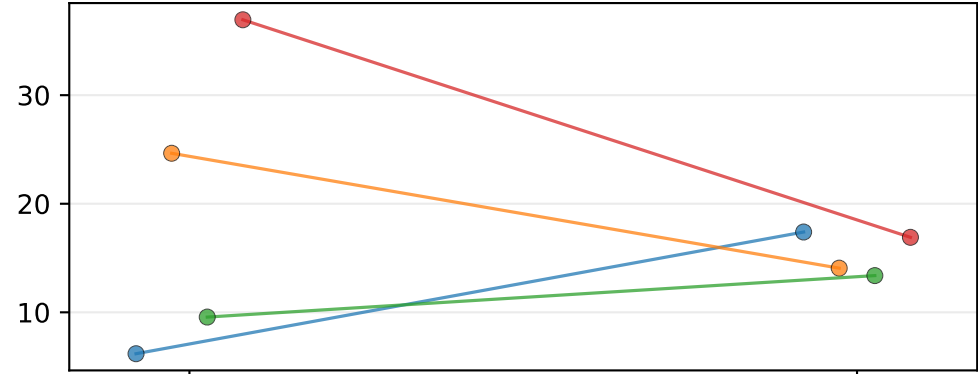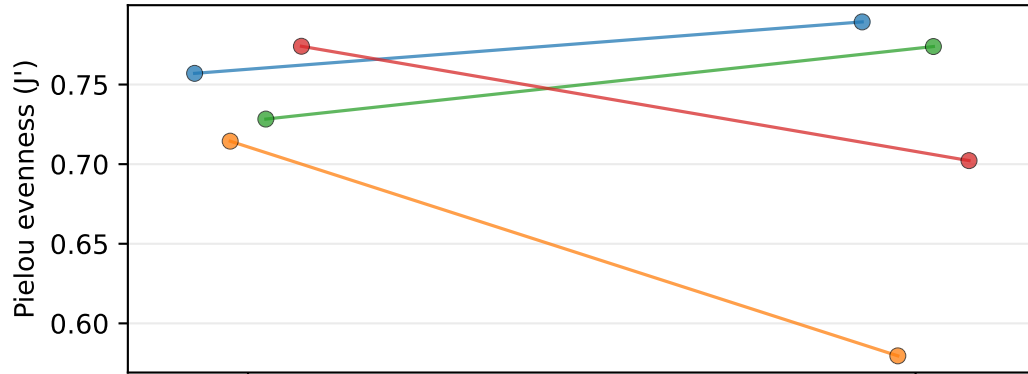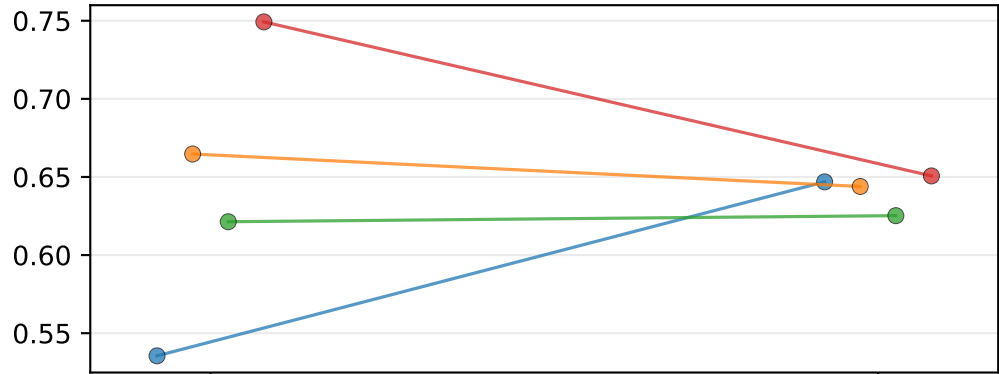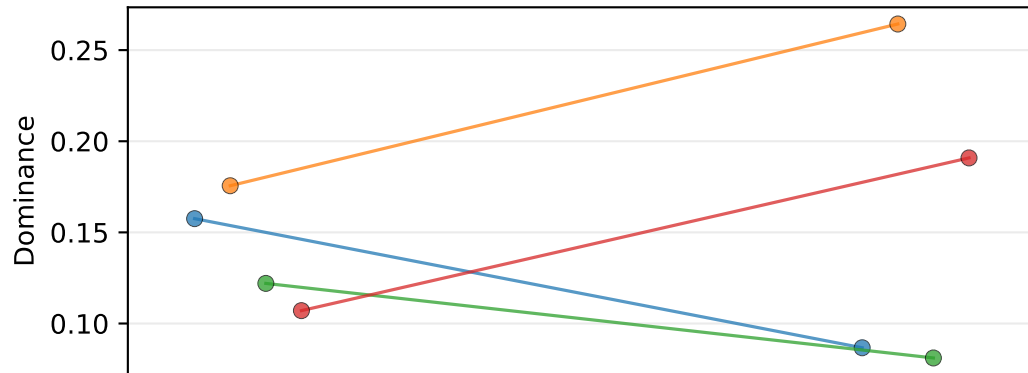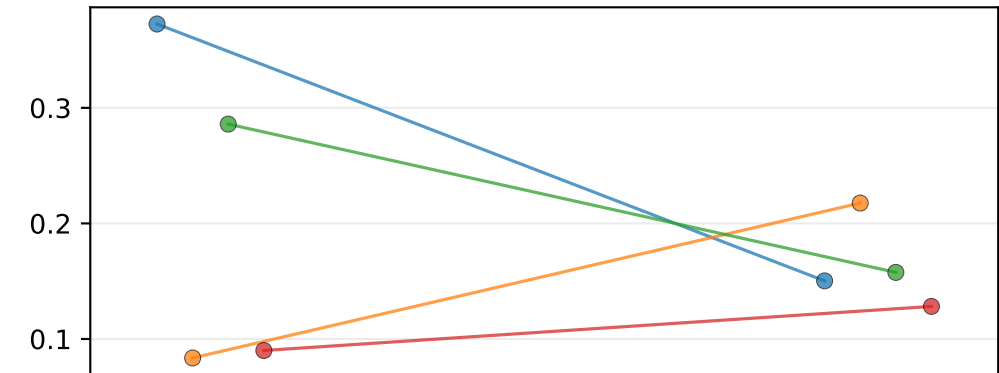

Supplement: S1 Fig — (PDF) [file pone.0352444.s005.pdf]
